# Efficacy and Safety of IncobotulinumtoxinA for the Simultaneous Treatment of Upper Facial Lines: A GRADE Assessed Systematic Review and Meta-analysis

**DOI:** 10.1093/asjof/ojag085

**Published:** 2026-05-09

**Authors:** Muhammad Tayyab Azam, Muhammad Hussain Azam, Muhammad Hassan Azam, Shahzad Ashraf, Muhammad Arif Khan, Hasibullah Aminpoor

## Abstract

**Background:**

Upper facial lines (UFLs), including glabellar frown lines (GFLs), horizontal forehead lines (HFLs), and lateral canthal lines (LCLs) are prevalent aesthetic concerns addressed with botulinum neurotoxin type A (BoNT-A). IncobotulinumtoxinA, a highly purified BoNT-A formulation, is utilized for simultaneous UFLs treatment; yet, comprehensive synthesized evidence is limited.

**Objectives:**

To systematically evaluate the efficacy and safety of IncobotulinumtoxinA compared with placebo for the simultaneous treatment of UFLs in adults, following PRISMA 2020 guidelines.

**Methods:**

Comprehensive searches of PubMed, Embase, Cochrane CENTRAL, ICTRP, and ClinicalTrials.gov were performed to identify randomized controlled trials (RCTs) evaluating IncobotulinumtoxinA for combined UFLs treatment. The primary outcome was ≥1-grade improvement on Day 30. Secondary outcomes included ≥2-grade improvement and Global Aesthetic Improvement Scale (GAIS) scores. Safety outcomes included adverse events (AEs), treatment-emergent adverse events (TEAEs), and headaches.

**Results:**

Three RCTs (n = 704) were included. Pooled analysis using random-effects model demonstrated a significant ≥1-grade improvement at Day 30 in IncobotulinumtoxinA compared with placebo across all regions: GFLs (RR 21.49, 95% CI: 9.89-46.73), HFLs (RR 19.17, 95% CI: 9.23-39.80), and LCLs (RR 11.68, 95% CI: 7.49-18.20), all *P* < .00001. A ≥ 2-grade improvement similarly favored treatment across all areas (RRs 76.26-115.04; all *P* < .00001). Investigator-assessed GAIS scores also improved significantly (MD 2.29, 95% CI: 2.11-2.46, *P* < .00001). Serious AEs were lower with IncobotulinumtoxinA (RR 0.14, *P* = .03). There was no significant difference in TEAEs and headaches from placebo.

**Conclusions:**

IncobotulinumtoxinA demonstrates statistically significant efficacy and a comparable safety profile vs placebo for simultaneous UFLs treatment. These findings support its use in aesthetic practice, warranting further long-term investigation.

**Level of Evidence: 1 (Therapeutic):**

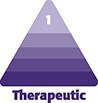

Facial aging, characterized by progressive development of wrinkles and lines in the upper face, is a major aesthetic concern affecting millions worldwide. The upper facial region, encompassing glabellar frown lines (GFLs), horizontal forehead lines (HFLs), and lateral canthal lines (LCLs), develops wrinkles from repeated muscle contractions during facial expressions. These dynamic wrinkles create an aged, fatigued, or severe appearance, potentially affecting self-perception and quality of life. Facial aging significantly affects self-confidence, social interactions, and life satisfaction.^1,2^ The demand for nonsurgical aesthetic interventions has increased substantially, with botulinum toxin type A (BoNT-A) becoming the leading procedure in the United States in 2022, accounting for 39% of all nonsurgical treatments with 3.9 million administrations.^3^

Botulinum neurotoxin type A is the gold standard for treating dynamic facial wrinkles, with well-established efficacy and safety.^4,5^ Botulinum toxin type A selectively blocks acetylcholine release at peripheral cholinergic nerve endings, temporarily inhibiting targeted facial muscle contraction.^4,5^ This temporary muscle relaxation reduces overlying wrinkles while preserving natural facial expressions. The treatment specifically targets dynamic wrinkles caused by repeated activity of the corrugator, procerus, frontalis, and orbicularis oculi muscles.^6^ The therapeutic effect typically appears within days and persists for several months.^7^ Decades of clinical use have established BoNT-A's favorable safety profile, with most adverse events (AEs) being mild, transient, and localized to injection sites.^8,9^

IncobotulinumtoxinA is a highly purified 150 kDa BoNT-A formulation without complexing proteins.^10,11^ Unlike other BoNT-A preparations having hemagglutinins and nontoxic proteins, IncobotulinumtoxinA consists solely of active neurotoxin. Complexing proteins, though not required for therapeutic effectiveness, can induce neutralizing antibody formation, potentially causing secondary treatment failure with repeated administrations.^12,13^ By eliminating complexing proteins, IncobotulinumtoxinA reduces foreign protein load per unit, potentially decreasing immunogenicity risk.^14^ This characteristic is especially relevant as patients seek treatment at younger ages and require multiple cycles over time.^15^ IncobotulinumtoxinA (Xeomin®, Merz Pharmaceuticals GmbH, Frankfurt, Germany) has received regulatory approval in over 80 countries, including the United States, European Union, Canada, and Japan, for both aesthetic and therapeutic indications.^16,17^ Notably, it remains the first and only botulinum neurotoxin type A granted FDA approval for the simultaneous treatment of Upper facial lines.^18^

Upper facial rejuvenation treatment has evolved from single-area to comprehensive multi-area approaches. While early studies and regulatory approvals focused on individual areas, particularly the glabellar region, current practice addresses multiple areas simultaneously.^19,20^ Simultaneous treatment of GFLs, HFLs, and LCLs has become standard practice.^17^ Combined treatment produces harmonized aesthetic outcomes, maintains facial proportions, and improves patient satisfaction compared with single-area treatment.^21,22^ Despite widespread clinical adoption, high-quality, placebo-controlled evidence for the efficacy and safety of simultaneous multi-area treatment has been lacking. Most regulatory trials focused on single indications, leaving a gap in the evidence base for the combined upper facial treatment approach.^23,24^

Recently, 3 Phase III, multicenter, randomized controlled trials have addressed this evidence gap by investigating IncobotulinumtoxinA for combined upper facial line treatment. A European multicenter study by Kerscher et al,^25^ ULTRA I (United States), and ULTRA II (Germany).^26^ These trials are the first placebo-controlled evidence for simultaneous treatment of GFLs, HFLs, and LCLs with IncobotulinumtoxinA. However, the drawbacks of existing trials, such as small sample sizes and short follow-up periods, underscore the need for a structured synthesis of existing data. While these constraints also apply to the present meta-analysis, pooling results allows for a more comprehensive and comparative assessment of efficacy and safety within the boundaries of current evidence. While a recent network meta-analysis by Li et al^27^ compared multiple BoNT-A formulations, its analysis was limited to patients treated for glabellar lines only. In contrast, this meta-analysis is the first to synthesize and consolidate the available evidence on IncobotulinumtoxinA vs placebo, assessing simultaneous treatment of glabellar, forehead, and lateral canthal lines, enabling comprehensive efficacy and safety evaluation, providing clinically meaningful effect estimates, and addressing practice-relevant treatment approaches not covered previously.

## METHODS

This systematic review and meta-analysis was conducted following the Preferred Reporting Items for Systematic Reviews and Meta-Analyses (PRISMA) 2020 guidelines. The PRISMA checklist is included in the Supplementary Figure 1.^28^ The protocol was registered with the International Prospective Register of Systematic Reviews under the reference number (CRD420251174687).

### Data Sources and Search Strategy

A comprehensive search was conducted using PubMed/MEDLINE, Cochrane CENTRAL, Embase, World Health Organization International Clinical Trials Registry Platform (WHO ICTRP), and ClinicalTrials.gov from inception till November 25, 2025 to identify all relevant studies. The reference lists of included articles and relevant reviews were also manually screened for additional studies. The search was conducted without limitations on language or publication date.

The search strategy was designed to be broad, encompassing a combination of MeSH terms and keywords related to the population, intervention, and comparator. The keywords for the search query were: (“Upper Facial Lines” OR “Glabellar Frown line” OR “Horizontal Forehead Line” OR “Lateral Canthal Line”) AND (“IncobotulinumtoxinA” OR “BoNT-A OR Xeomin OR NT 201”) AND (“Placebos”). A detailed search strategy for each database is provided in Supplementary Table 1.

### Study Selection and Eligibility Criteria

All identified records were first imported into EndNote reference software, version X8.1 (Clarivate Analytics), for initial deduplication, then uploaded into Rayyan.ai for systematic screening.^29,30^ Two reviewers (M.T.A., M.H.A.) independently screened the titles and abstracts of the remaining records for potential eligibility. Full texts of selected articles were retrieved and assessed against predefined inclusion criteria. Disagreements were resolved through consultation with a third reviewer (S.A.).

The inclusion criteria were defined using the Population, Intervention, Control, and Results (PICOS) methodology for systematic reviews and meta-analyses, where (*P*) denotes trials involving adult participants (aged ≥18 years) with moderate-to-severe upper facial lines, (I) denotes IncobotulinumtoxinA administered simultaneously in all UFLs regions, (C) denotes placebo, (O) denotes safety and efficacy outcomes, and (S) denotes randomized controlled trials.

Exclusion criteria were the following: (i) non-randomized controlled trials (RCTs), systematic reviews, and meta-analyses; (ii) nonindependent RCTs such as extension phases, and post-hoc analyses; (iii) studies with wrong comparator (eg, comparing different dilutions of incobotulinumtoxinA without placebo control); (iv) duplicate publications of the same trial including its trial registry.

### Data Extraction

Data extraction was independently performed by 2 authors (M.H.A., S.A.) on a pre-piloted Microsoft Excel sheet, with discrepancies resolved by consulting the third author (M.T.A.). Extracted information included study identifiers (first author, year, country, phase), study design, baseline patient characteristics (age, sex), intervention details, comparator details, efficacy outcomes and safety outcomes. The primary efficacy outcome was ≥1-Grade Improvement on MAS (Merz Aesthetics Scale; defined in the Supplementary Figure 2) at Day 30 for each treated facial area. Secondary efficacy outcomes were Investigator and Participant assessed Global Aesthetic Improvement Scale (GAIS) scores, and ≥2-Grade Improvement on MAS at Day 30. Safety outcomes were incidence of treatment-emergent AEs, serious AEs, and AEs of special interest including ptosis and headache. For categorical outcomes, data on the number of events and total sample size were extracted; for continuous outcomes, mean values, standard deviations, and sample sizes were collected.

### Quality Assessment

Two independent reviewers (M.A.K., M.H.A.) assessed the risk of bias using the Cochrane Risk of Bias 2.0 (RoB 2.0) tool.^31^ This tool evaluates bias across 5 domains: bias from the randomization process, bias from deviations from intended interventions, bias from missing outcome data, bias in outcome measurement, and bias in reported results. Each domain was graded as “low risk,” “some concerns,” or “high risk.” Disagreements were resolved through discussion with a third reviewer.

### Statistical Analysis

All statistical analyses were conducted using Review Manager (RevMan, version 5.4; Cochrane Collaboration, Copenhagen, Denmark). For dichotomous outcomes, risk ratios (RR) with 95% CIs were calculated. For continuous outcomes, mean differences with 95% CIs were calculated. Given the anticipated clinical heterogeneity across geographic populations and trial sites, a random-effects model (DerSimonian-Laird method) was used for all pooled analyses.^32^ Results were graphically displayed as forest plots. A *P*-value of <.05 was considered statistically significant. Statistical heterogeneity was assessed using the I^2^ statistic, with thresholds of <20%, 25% to 50%, and >50% indicating low, moderate, and high heterogeneity, respectively.^33^

### Certainty of Evidence (GRADE Assessment)

The certainty of evidence for each primary outcome was assessed using the Grading of Recommendations, Assessment, Development, and Evaluations (GRADE) framework.^34^ The GRADE approach evaluates the quality of evidence based on 5 domains: risk of bias, inconsistency, indirectness, imprecision, and publication bias. The overall certainty was rated as high, moderate, low, or very low. Grading of Recommendations, Assessment, Development, and Evaluations certainty reporting of the main outcomes is presented in Supplementary Table 2.

## RESULTS

### Study Selection

**Figure 1.**
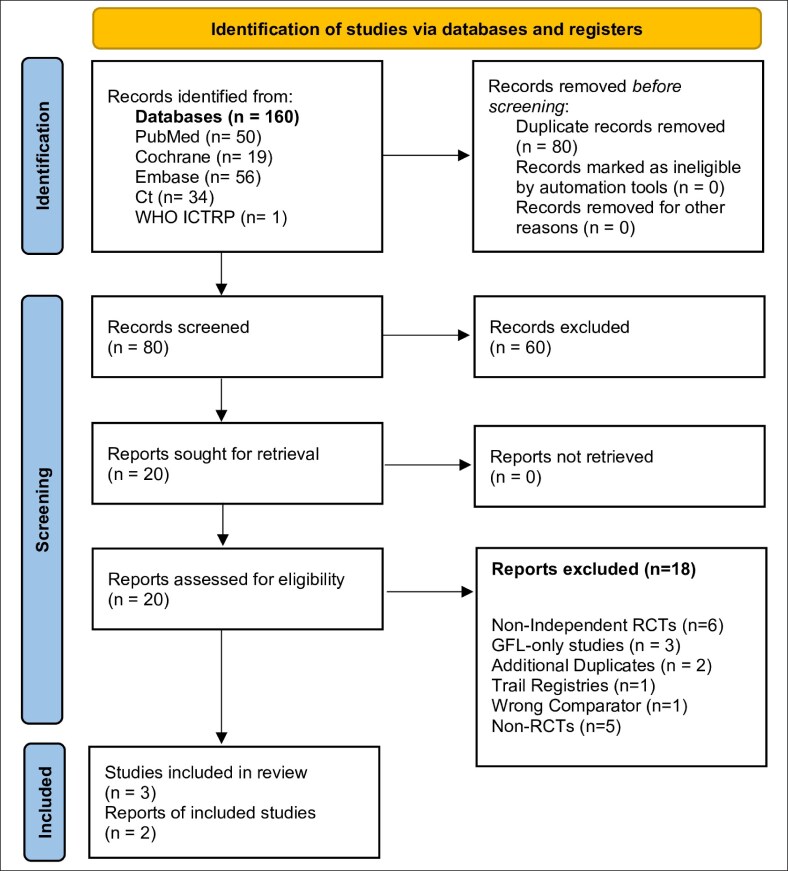
PRISMA flow diagram represents screening process.

The study selection was performed following PRISMA guidelines. The initial search across 5 databases PubMed (n = 51), Cochrane (n = 19), Embase (n = 56), ClinicalTrials.gov (n = 34), and WHO ICTRP (n = 1) yielded 161 records. After removal of 81 duplicates, 80 unique records were screened by title and abstract, resulting in the exclusion of 60 studies. Twenty full-text articles were assessed for eligibility, of which 18 were excluded for the following reasons: nonindependent RCTs (n = 6), studies limited to glabellar lines (n = 3), additional duplicates (n = 2), trial registries (n = 1), inappropriate comparator (n = 1), and non-RCT designs (n = 5). Ultimately, 3 studies met the predefined inclusion criteria and were incorporated into the meta-analysis.^25,26^ The detailed selection process is illustrated in Figure 1.

### Study and Patient Characteristics

Our meta-analysis included 3 Phase III, multicenter, placebo-controlled randomized controlled trials, reported in 2 publications, conducted in the United States and Europe, encompassing a total of 704 participants. The mean age ranged from 44.8 to 47.8 years, and most participants were female (79.9%-89.5%). Baseline assessments showed a substantial proportion of participants had moderate to severe facial line severity. A detailed baseline characteristics table is presented in Table 1.

**Table 1.** Baseline Characteristics of Included Studies

Baseline characteristics	ULTRA-I	ULTRA-II	Kerscher et al phase 3 study
Country	12 sites across United States	12 sites across Germany	10 sites located across Germany, France, and the United Kingdom
Study type	Phase III prospective, multicenter, international, placebo-controlled RCT	Phase III prospective, multicenter, international, placebo-controlled RCT	Phase III prospective, multicenter, international, placebo-controlled RCT

Intervention	Group Pa (N = 91)	Group Ua (N = 179)	Group G&Ha (N = 92)	Group Pa (N = 94)	Group Ua (N = 184)	Group La (N = 90)	IncobotulinumtoxinA (N = 105)	Placebo (N = 51)	
Age mean SD	47.5 [13.0]	47.2 [13.2]	47.8 [13.5]	44.8 [9.15]	46.0 [9.92]	45.9 [9.88]	47.4 ± 10.1	47.5 ± 8.4	
Female sex n (%)	79 (86.8)	154 (86.0)	75 (81.5)	84 (89.4)	147 (79.9)	72 (80.0)	94 (89.5%)	41 (80.4%)	
Moderate (MAS score = 2) baseline severity, investigator assessed (GFLs)	39.60%	39.10%	40.20%	44.70%	45.70%	47.80%	32 (30.5)	13 (25.5)	
Moderate (MAS score = 2) baseline severity, investigator assessed (HFLs)	33.00%	30.7%	26.1%	27.70%	29.30%	28.90%	18 (17.1)	13 (25.5)	
Moderate (MAS score = 2) baseline severity, investigator assessed (right LCLs)	39.60%	45.80%	44.60%	50.00%	49.50%	36.70%	Lateral Canthal Lines (LCLs)		
							28 (26.7)		14 (27.5)
Moderate (MAS score = 2) baseline severity, investigator assessed (left LCLs)	39.60%	45.80%	44.60%	50.00%	49.50%	36.70%	N/A		N/A
Severe (MAS score = 3) baseline severity, investigator assessed (GFLs)	60.40%	60.90%	59.80%	55.30%	54.30%	52.20%	73 (69.5)		38 (74.5)
Severe (MAS score = 3) baseline severity, investigator assessed (HFLs)	67.00%	69.30%	73.90%	72.30%	70.70%	71.10%	87 (82.9)		38 (74.5)
Severe (MAS score = 3) baseline severity, investigator assessed (right LCLs)	60.40%	54.20%	55.40%	50.00%	50.50%	63.30%	Lateral canthal lines (LCLs)		
							76 (72.4)		37 (72.5)

GFLs, glabellar frown lines; Group G&Ha, only GFLs and HFLs treated with incobotulinumtoxinA; Group La, only LCLs treated with IncobotulinumtoxinA; Group Pa, placebo group; Group Ua, upper facial lines (a combination of GFLs HFLs and LCLs) treated with IncobotulinumtoxinA; HFLs, horizontal forehead lines; LCLs, lateral canthal lines; LCLs, Lateral canthal lines; MAS, Merz Aesthetic Scale; N, Number of participants; RCT, Randomized Controlled Trials.

### Risk of Bias Assessment

Three studies were evaluated for both efficacy and safety outcomes. Overall, most domains across these studies were judged to be at low risk of bias, reflecting generally high methodological quality. However, Kerscher et al (2015) raised some concerns in Domain 3, which addresses missing outcome data, for both efficacy and safety outcomes, as shown in Supplementary Figures 3 and 4. This was due to incomplete reporting, which could potentially affect the reliability of results. Despite this limitation, the remaining domains in Kerscher et al (2015) and the other 2 studies were considered robust, supporting the overall credibility of the findings.

### Primary Outcomes

#### ≥1-Grade Improvement at Day 30

**Figure 2.**
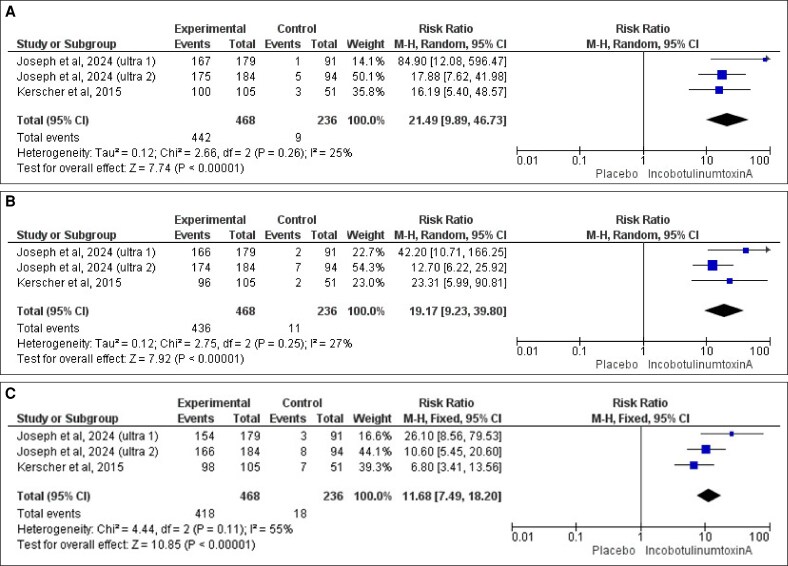
Forest plot of the ≥1-grade improvement on day 30 for (A) Glabellar Frown Lines (GFLs), (B) Horizontal Forehead Lines (HFLs), and (C) Lateral Canthal Lines (LCLs) comparing IncobotulinumtoxinA with placebo. Effect measure: risk ratio (RR) with 95% CIs, analyzed using a random-effects model.

Three randomized controlled trials (RCTs) encompassing a total of 704 participants reported results for ≥1-grade improvement at Day 30 across all facial regions. IncobotulinumtoxinA demonstrated statistically significant results compared with placebo. Significant improvements were observed for glabellar frown lines (RR 21.49, 95% CI: 9.89-46.73; *P* < .00001; I^2^ = 25%), horizontal forehead lines (RR 19.17, 95% CI: 9.23-39.80; *P* < .00001; I^2^ = 27%), and lateral canthal lines (RR 11.68, 95% CI: 7.49-18.20; *P* < .00001; I^2^ = 55%), as reported in Figure 2A-C, respectively. Overall, these findings suggest that IncobotulinumtoxinA may offer a better effect over placebo with low-to-moderate heterogeneity.

### Secondary Outcomes

#### ≥2-Grade Improvement at Day 30

**Figure 3.**
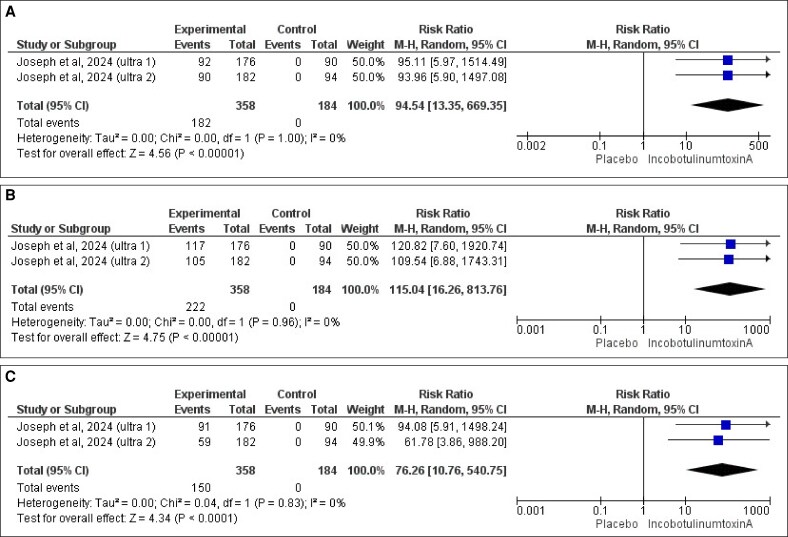
Forest plot of the ≥2-grade improvement on day 30 for (A) Glabellar Frown Lines (GFLs), (B) Horizontal Forehead Lines (HFLs), and (C) Lateral Canthal Lines (LCLs) comparing IncobotulinumtoxinA with placebo. Effect measure: risk ratio (RR) with 95% CIs, analyzed using a random-effects model.

Two RCTs (n = 542) assessed the ≥2-grade improvement on Day 30. IncobotulinumtoxinA demonstrated better treatment effect compared with placebo across all facial regions. Significant improvements were observed for glabellar frown lines (RR 94.54, 95% CI: 13.35-669.35; *P* < .00001; I^2^ = 0%), horizontal forehead lines (RR 115.04, 95% CI: 16.26-813.76; *P* < .00001; I^2^ = 0%), and lateral canthal lines (RR 76.26, 95% CI: 10.76-540.75; *P* < .00001; I^2^ = 0%), as reported in Figure 3A-C, respectively. No heterogeneity observed in the pooled analysis suggesting consistent findings across these RCTs.

#### Investigator-Assessed Global AestheticImprovement Scale (GAIS) Scores

**Figure 4.**
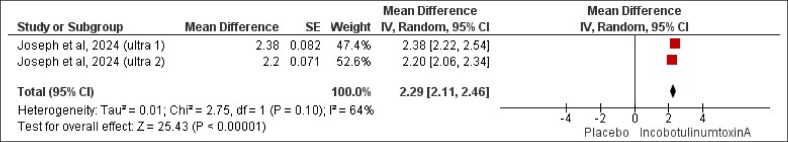
Forest plot of the investigator-assessed GAIS (Global Aesthetic Improvement Scale) scores comparing IncobotulinumtoxinA with placebo. Effect measure: mean difference (MD) with 95% CIs, analyzed using a random-effects model.

Two RCTs (n = 543) reported the results for the investigator-assessed GAIS (Global Aesthetic Improvement Scale) scores. IncobotulinumtoxinA significantly increased the score compared with placebo. The pooled Mean Difference was 2.29 (95% CI: 2.11-2.46; *P* < .00001; I^2^ = 64%), as reported in Figure 4.

#### Participant-Assessed GAIS Scores

**Figure 5.**
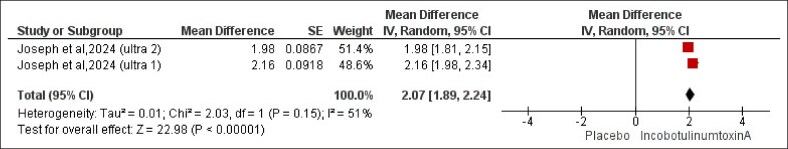
Forest plot of the participant-assessed GAIS (Global Aesthetic Improvement Scale) scores comparing IncobotulinumtoxinA with placebo. Effect measure: mean difference (MD) with 95% CIs, analyzed using a random-effects model.

Two RCTs (n = 543) reported the results for the participant-assessed GAIS (Global Aesthetic Improvement Scale) scores. IncobotulinumtoxinA significantly increased the score compared with placebo. The pooled Mean Difference was 2.07 (95% CI: 1.89-2.24; *P* < .00001; I^2^ = 51%), as reported in Figure 5.

#### Investigator-Assessed Merz Aesthetic Scale 0/1 Responders at Day 30

**Figure 6.**
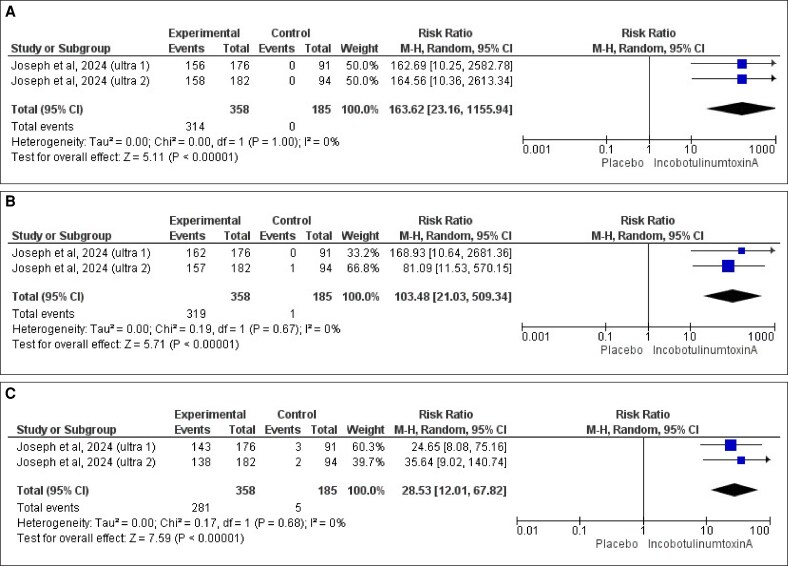
Forest plot of the investigator-assessed MAS (Merz Aesthetics Scale) 0/1 responders at Day 30 for (A) Glabellar Frown Lines (GFLs), (B) Horizontal Forehead Lines (HFLs), and (C) Lateral Canthal Lines (LCLs) comparing IncobotulinumtoxinA with placebo. Effect measure: risk ratio (RR) with 95% CIs, analyzed using a random-effects model.

Two RCTs (n = 543) reported the results for investigator-assessed MAS 0/1 responders. IncobotulinumtoxinA significantly increased responder rates compared with placebo. The pooled RRs were 163.62 (95% CI: 23.16-1155.94; *P* < .00001; I^2^ = 0%) for glabellar lines, 103.48 (95% CI: 21.03-509.34; *P* < .00001; I^2^ = 0%) for horizontal forehead lines, and 28.53 (95% CI: 12.01-67.82; *P* < .00001; I^2^ = 0%) for lateral canthal lines, as reported in Figure 6A-C, respectively.

#### Participant-Assessed Merz Aesthetic Scale 0/1 Responders at Day 30

**Figure 7.**
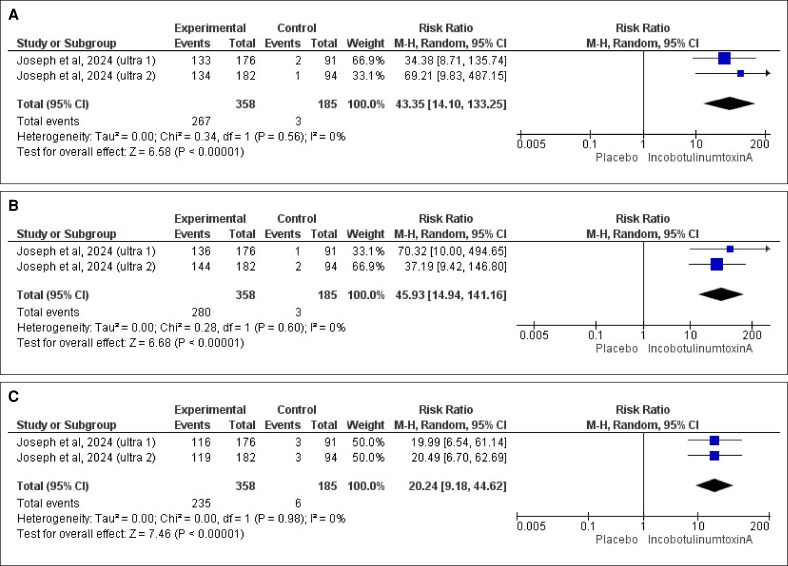
Forest plot of the participant-assessed MAS (Merz Aesthetics Scale) 0/1 responders at day 30 for (A) Glabellar Frown Lines (GFLs), (B) Horizontal Forehead Lines (HFLs), and (C) Lateral Canthal Lines (LCLs) comparing IncobotulinumtoxinA with placebo. Effect measure: risk ratio (RR) with 95% CIs, analyzed using a random-effects model.

Two RCTs (n = 543) reported results for participant-assessed MAS 0/1 responders. IncobotulinumtoxinA demonstrated significantly higher responder rates than placebo. The pooled RRs were 43.35 (95% CI: 14.10-133.25; *P* < .00001; I^2^ = 15%) for glabellar lines, 45.93 (95% CI: 14.94-141.16; *P* < .00001; I^2^ = 12%) for horizontal forehead lines, and 20.24 (95% CI: 9.18-44.62; *P* < .00001; I^2^ = 21%) for lateral canthal lines, as reported in Figure 7A-C, respectively. Low heterogeneity shows consistent findings across the outcome.

### Safety Outcomes

#### Serious Adverse Events

**Figure 8.**
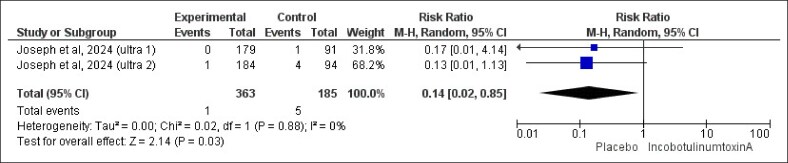
Forest plot of the serious adverse events comparing IncobotulinumtoxinA with placebo. Effect measure: risk ratio (RR) with 95% CIs, analyzed using a random-effects model.

Two RCTs (n = 548) were included in the pooled analysis of serious adverse events (SAEs). SAEs were rare overall, with 1/363 (0.3%) in the incobotulinumtoxinA groups vs 5/185 (2.7%) in the placebo groups across both trials. The pooled analysis showed a statistically lower incidence in the incobotulinumtoxinA group (RR 0.14, 95% CI: 0.02-0.85; *P* = .03; I^2^ = 0%), as reported in Figure 8. Importantly, none of the reported SAEs were deemed treatment-related by study investigators, supporting a favorable safety profile.

#### Any Adverse Events and Headache

**Figure 9.**
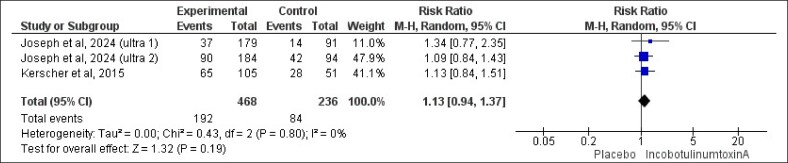
Forest plot of any adverse events comparing IncobotulinumtoxinA with placebo. Effect measure: risk ratio (RR) with 95% CIs, analyzed using a random-effects model.

Three RCTs (n = 704) contributed to the analysis of any AEs and headaches. No statistically significant differences were observed between IncobotulinumtoxinA and placebo: any AEs (RR 1.13, 95% CI: 0.94-1.37; *P* = .19; I^2^ = 19%) and headache (RR 2.05, 95% CI: 0.51-8.25; *P* = .31; I^2^ = 55%), as reported in Figure 9 and Supplementary Figure 5, respectively.

#### Other Safety Outcomes

**Figure 10.**
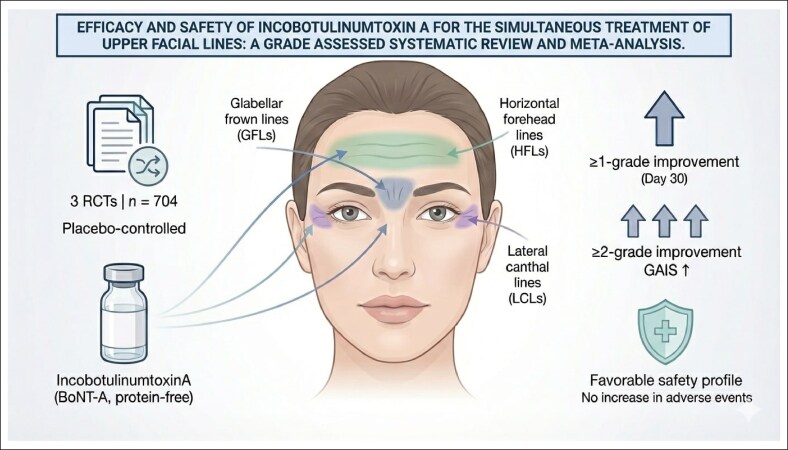
The graphical central illustration summarizing the key efficacy and safety findings.

Two RCTs (n = 548) contributed to treatment-emergent adverse events (TEAEs), COVID-19–related TEAEs, COVID-19 infection, and brow ptosis. No significant differences were observed for TEAEs (RR 1.63, 95% CI: 0.93-2.86; *P* = .09; I^2^ = 0%), COVID-19–related TEAEs (RR 0.66, 95% CI: 0.15-2.84; *P* = .58; I^2^ = 0%), COVID-19 infection (RR 0.64, 95% CI: 0.13-3.27; *P* = .59; I^2^ = 0%), or brow ptosis (RR 2.43, 95% CI: 0.28-21.18; *P* = .42; I^2^ = 0%), as shown in Supplementary Figures 6-9, respectively. The graphical central illustration summarizing the main findings is presented in Figure 10.

## DISCUSSION

This meta-analysis of 3 randomized controlled trials evaluating incobotulinumtoxinA for upper facial lines suggests clinically meaningful improvements across all treated regions including glabellar frown lines, horizontal forehead lines, and lateral canthal lines. IncobotulinumtoxinA demonstrated an increased likelihood of achieving ≥1-grade and ≥2-grade improvements compared with placebo, with similarly robust results for MAS 0/1 responder rates using both investigator- and participant-assessed scores. GAIS outcomes further added to the evidence of high patient-perceived aesthetic benefit. Safety findings demonstrated no increase in overall AEs, headaches, TEAEs, or brow ptosis compared to placebo. Additionally, serious AEs were less frequent with IncobotulinumtoxinA. Moreover, Low between-study heterogeneity supports the reliability and reproducibility of these effects. Overall, the pooled evidence shows that IncobotulinumtoxinA is a highly effective and well-tolerated therapy for comprehensive upper facial line treatment.

The efficacy findings from our meta-analysis are aligned with the results reported in the original trials that contributed to the pooled data. In the pivotal 2015 Phase III study, subjects treated with 54-64 U of incobotulinumtoxinA demonstrated Day 30 responder rates of 84.5% for glabellar frown lines, 70.9% for horizontal forehead lines, and 64.1% for lateral canthal lines, compared with 0%-2% in placebo.^25^ Similarly, the ULTRA I & II trials (2024) also reported significant improvements across all investigator- and participant-assessed endpoints (*P* < .0001).^26^ These individual trial results form the basis of the pooled evidence synthesized in our meta-analysis and demonstrate consistent treatment effects across different study populations. Regarding safety, our analysis is consistent with the safety findings of these trials. For example, the 2015 Phase III study reported only 2 cases of mild, transient eyelid ptosis among 105 participants, with no serious systemic AEs. Larger pooled analyses, comprising over 1300 subjects across multiple aesthetic indications, reported treatment-related AEs ranging from 5.4% to 22.9%, with headache being the most common (11.4% in upper-face single-dose studies); yet, no serious treatment-related events occurred.^35^ Similarly, the 2024ULTRA I & II trials found no new safety concerns during the 120-day main study period or throughout the 8-month open-label extension (OLEX).^26,36^ Collectively, these data support the favorable tolerability profile of incobotulinumtoxinA when used for simultaneous upper facial line treatment.

When comparing IncobotulinumtoxinA with other botulinum toxin A, the evidence suggests that it has a similar efficacy with well-tolerated results for aesthetic purposes, particularly for glabellar frown lines. In randomized, double-blind trials, IncobotulinumtoxinA and OnabotulinumtoxinA administered at equivalent units (20 U) produced comparable responder rates at Day 28-30 (95%-96%) and high patient satisfaction, with no significant differences in AEs.^37,38^ Some studies suggest that incobotulinumtoxinA may have a faster onset and longer duration of effect compared with onabotulinumtoxinA and other botulinum toxins. However, these differences do not translate into clinically superior efficacy over onabotulinumtoxinA or other neurotoxins in upper facial line treatment.^7^

However, preclinical pharmacologic studies reveal that the labeled units of different formulations cannot be directly equivalent; for example, enzymatic activity and neuromuscular blockade can vary between products. This underscores the importance of tailoring dosing based on clinical response rather than assuming strict unit-to-unit interchangeability.^39^ Taken together, these findings support IncobotulinumtoxinA as a reliable, effective, and well-tolerated alternative to other botulinum toxin A products, while suggesting individualized dosing, particularly when treating multiple upper-face regions simultaneously.

Beyond what is already known from comparisons with onabotulinumtoxinA, an expanding body of research shows that IncobotulinumtoxinA performs just as reliably as other commonly used botulinum toxin type A formulations. A comprehensive systematic review found that IncobotulinumtoxinA, onabotulinumtoxinA, and abobotulinumtoxinA achieve very similar aesthetic outcomes, with no meaningful differences in wrinkle reduction across studies highlighting how comparable these products truly are in clinical practice.^40^ This is supported by split-face and split-region studies, where IncobotulinumtoxinA and abobotulinumtoxinA consistently softened dynamic lines such as crow's feet and produced high levels of patient satisfaction, again with no major distinctions between the 2 treatments.^41^ More recent randomized trials, including those evaluating prabotulinumtoxinA, suggest that some formulations may vary slightly in how quickly they take effect or how long they last; however, every product tested still delivered significant patient-reported improvements for up to 90 days.^42^ Taken together, these findings show that while IncobotulinumtoxinA may not outperform other toxins in every single measure, it remains fully comparable to other leading BoNT-A options. This gives clinicians the flexibility to choose the most appropriate product for each patient while remaining confident in its reliable efficacy and reassuring safety profile.^42^

### Strengths and Limitations

This comprehensive systematic review and meta-analysis analyzed phase III, randomized, placebo-controlled trials. Included studies had a sample of participants from clinical sites across multiple countries, including the United States, Germany, and the UK. The studies followed rigorous measures (eg, Merz Aesthetics Scale [MAS]) with trained investigators to assess the severity of the outcomes. The outcomes were clear, well-defined, and clinically relevant. The protocols administered to the included participants were consistent, detailed, and standardized, which enhances the reproducibility of the findings. Included studies assessed most of the upper facial regions simultaneously (GFLs, HFLs, and LCLs), which implies real-world aesthetics. The studies included a toxin-naïve and previously treated, and participants with different skin types, enhancing the applicability to a broader aesthetic population. The drawbacks of this meta-analysis are the inclusion of a limited number of studies with a modest sample size (n = 704) and a predominantly female participant pool, which limits the statistical power and generalizability of the results to a broader population. The inclusion criteria were restrictive, as all the trials exclusively enrolled patients with moderate-to-severe wrinkle types with no prior dermatologic conditions, no laser or surgical procedures, or any structural abnormalities, which does not correlate with real-world aesthetics. Despite investigators being blinded, the use of subjective investigator scoring (eg, MAS and GAIS) may introduce potential bias.

### Future Directions

Future research in this area should focus on refining and contextualizing the formally evaluated and recently regulatorily approved paradigm of simultaneous multiregional upper facial treatment. There is a need for large, more diverse cohorts, including varying demographics, to better represent global aesthetic populations. Future trials should also involve common comorbid conditions, prior aesthetic procedures, and variable factors (eg, smokers, sun damage) to reflect routine clinical settings. Future trials should evaluate incobotulinumtoxinA in multimodal aesthetic strategies and should reduce the artificial restrictions imposed by study protocols to improve real-world relevance. Future trials should involve artificial intelligence-assisted software and standardized injection mapping to reduce inconsistencies in practitioner techniques. There is also a need for more robust patient-reported outcome measures, including assessment of aesthetic satisfaction, psychosocial benefits, and quality of life, to address patient-centered treatment effects. The favorable findings support the consideration of incobotulinumtoxinA in clinical practice; however, additional rigorous, well-designed trials can still contribute to the literature by enhancing the generalizability of the treatment approach, strengthening the evidence base, and solidifying its role in clinical settings.

## CONCLUSIONS

This systematic review and meta-analysis confirms that IncobotulinumtoxinA provides consistent and clinically meaningful improvements in wrinkle severity across all upper facial regions compared with placebo, supported by statistically significant effects across multiple primary and secondary endpoints. Many of the adverse effects were mild and self-limiting, thereby exhibiting a favorable safety profile. The superiority of the active treatment (ie, incobotulinumtoxinA) was supported by both investigator-assessed and patient-reported outcomes, showing concordance between clinical improvements and subjective patient satisfaction, reinforcing its applicability in clinical settings.

## Supplemental Material

This article contains supplemental material located online at https://doi.org/10.1093/asjof/ojag085.

## Supplementary Material

ojag085_Supplementary_Data
