# Supplementary material for: Efficacy and Safety of IncobotulinumtoxinA for the Simultaneous Treatment of Upper Facial Lines: A GRADE Assessed Systematic Review and Meta-analysis
Source: Aesthet Surg J Open Forum. 2026 May 9;8:ojag085. doi: 10.1093/asjof/ojag085 (PMC13253580; doi:10.1093/asjof/ojag085)
Supplement: ojag085_Supplementary_Data [file ojag085_supplementary_data.zip › Supplementary Tables.docx]

**Supplementary Table 1:** Detailed search strategies used for each database

| **DATABASE** | **SEARCH STRATEGY** | **SEARCH RESULTS** |
| --- | --- | --- |
| **Pubmed** | **(((((((Upper Facial Lines) OR (Glabellar Frown Line)) OR (Horizontal Forehead Line)) OR (GFL)) OR (HFL)) OR (Lateral Canthal Line)) OR (LCL)) AND (((((("incobotulinumtoxinA" [Supplementary Concept]) OR (BoNT-A)) OR (NT 201)) OR (NT201)) OR (Xeomin)) OR (bocouture)) AND Placebo** | **51** |
| **Clinical trails.gov** | **Condition/disease: Upper facial Lines OR Glabellar OR Horizontal OR Lateral Canthal OR GFL OR HFL OR LCL**  **Other terms:**  **Intervention/treatment: IncobotulinumtoxinA OR BoNT-A OR NT 201 OR NT201 OR NT-201 OR Xeomin OR bocouture** | **34** |
| **Cochrane** | **ID Search Hits**  **#1 Upper Facial Lines 138**  **#2 Glabellar Frown Line 78**  **#3 GFL 44**  **#4 Horizontal Forehead Line 4**  **#5 HFL 78**  **#6 Lateral Canthal Line 20**  **#7 LCL 146**  **#8 #1 OR #2 OR #3 OR #4 OR #5 OR #6 OR #7 443**  **#9 Incobotulinumtoxina 340**  **#10 BoNT-A 35**  **#11 NT 201 275**  **#12 NT201 14**  **#13 NT-201 89**  **#14 Xeomin 244**  **#15 bocouture 11**  **#16 #9 OR #10 OR #11 OR #12 OR #13 OR #14 OR #15 723**  **#17 Placebo 419130**  **#18 #8 AND #16 AND #17 19** | **19** |
| **Embase** | **'upper facial lines' AND ('botulinum toxin a'/exp OR 'btxa' OR 'bont a' OR 'bont a ds' OR 'bont serotype a' OR 'clostridium botulinum neurotoxin a' OR 'clostridium botulinum neurotoxin type a' OR 'clostridium botulinum type a neurotoxin' OR 'abobotulinum toxin a' OR 'abobotulinumtoxin a' OR 'abobotulinumtoxina' OR 'agn 151607' OR 'agn151607' OR 'alluzience' OR 'ant 1207' OR 'ant 1401' OR 'ant 1403' OR 'ant1207' OR 'ant1401' OR 'ant1403' OR 'azzalure' OR 'bocouture' OR 'boe-tox' OR 'botox' OR 'botox (100 u) injection' OR 'botox (oculinum)' OR 'botox 100e' OR 'botox a' OR 'botox cosmetic' OR 'botulift' OR 'botulin a' OR 'botulin toxin a' OR 'botulinium a toxin' OR 'botulinum a exotoxin' OR 'botulinum a toxin' OR 'botulinum neurotoxin a' OR 'botulinum neurotoxin type a' OR 'botulinum toxin a' OR 'botulinum toxin type a' OR 'botulinum toxins, type a' OR 'clostridium botulinum a toxin' OR 'clostridium botulinum endotoxin' OR 'clostridium botulinum toxin type a' OR 'cnt 52120' OR 'cnt52120' OR 'corabotase' OR 'cunox' OR 'daxibotulinum toxin a' OR 'daxibotulinumtoxin a' OR 'daxibotulinumtoxin a lanm' OR 'daxibotulinumtoxina' OR 'daxibotulinumtoxina lanm' OR 'daxibotulinumtoxina-lanm' OR 'daxxify' OR 'dtx 021' OR 'dtx021' OR 'dwp 450' OR 'dwp450' OR 'dyslor' OR 'dysport' OR 'evabotulinum toxin a' OR 'evabotulinumtoxin a' OR 'evabotulinumtoxina' OR 'evosyal' OR 'gemibotulinum toxin a' OR 'gemibotulinumtoxin a' OR 'gemibotulinumtoxina' OR 'gsk 1358820' OR 'gsk1358820' OR 'hg 102' OR 'hg102' OR 'hu 014' OR 'hu014' OR 'hutox' OR 'incobotulinum toxin a' OR 'incobotulinumtoxin a' OR 'incobotulinumtoxina' OR 'ipn 10200' OR 'ipn 59011' OR 'ipn10200' OR 'ipn59011' OR 'jeuveau' OR 'letibotulinum toxin a' OR 'letibotulinumtoxin a' OR 'letibotulinumtoxina' OR 'letibotulinumtoxina wlbg' OR 'letibotulinumtoxina-wlbg' OR 'letybo' OR 'liztox' OR 'lp 09' OR 'lp09' OR 'meditoxin' OR 'mt 10107' OR 'mt 10109' OR 'mt10107' OR 'mt10109' OR 'nabota' OR 'neuronox' OR 'nivobotulinum toxin a' OR 'nivobotulinumtoxin a' OR 'nivobotulinumtoxina' OR 'nt 201' OR 'nt201' OR 'nuceiva' OR 'oculinum' OR 'onabotulinum toxin a' OR 'onabotulinumtoxin a' OR 'onabotulinumtoxina' OR 'onaclostox' OR 'pm 12759' OR 'pm12759' OR 'prabotulinum toxin a' OR 'prabotulinumtoxin a' OR 'prabotulinumtoxin a xvfs' OR 'prabotulinumtoxina' OR 'prabotulinumtoxina xvfs' OR 'prabotulinumtoxina-xvfs' OR 'prosigne' OR 'purtox' OR 'qm 1114' OR 'qm1114' OR 'relabotulinum toxin a' OR 'relabotulinumtoxin a' OR 'relabotulinumtoxina' OR 'relfydess' OR 'reloxin' OR 'rt 002' OR 'rt002' OR 'rtt 150' OR 'rtt150' OR 'vistabel' OR 'vistabex' OR 'xeomeen' OR 'xeomin')** | **56** |
| **WHO ICTRP** | **Condition/disease: Upper Facial lines OR Glabellar Frown Line OR Horizontal Frown Line OR GFL OR HFL OR Lateral Canthal Line OR LCL**  **Intervention/treatment: IncobotulinumtoxinA OR BoNT-A OR NT 201 OR NT201 OR NT-201 OR Xeomin OR bocouture** | **01** |

Databases. No of results

Ct: 34

Cochrane: 19

PubMed: 51

Embase: 56

WHO ICTRP: 01

**Total Search Results:161**

**Supplementary Table 2:** GRADE certainty reporting of main outcomes

| **IncobotulinumtoxinA compared to Placebo for Upper Facial Lines**  **Bibliography:** | | | | | | | | | | | |
| --- | --- | --- | --- | --- | --- | --- | --- | --- | --- | --- | --- |
| **Certainty assessment** | | | | | | | **Summary of findings** | | | | |
| **Participants (studies) Follow-up** | **Risk of bias** | **Inconsistency** | **Indirectness** | **Imprecision** | **Publication bias** | **Overall certainty of evidence** | **Study event rates (%)** | | **Relative effect (95% CI)** | **Anticipated absolute effects** | |
|  |  |  |  |  |  |  | **With Placebo** | **With IncobotulinumtoxinA** |  | **Risk with Placebo** | **Risk difference with IncobotulinumtoxinA** |
| **≥1-Grade Improvement at Day 30 for GFLs** | | | | | | | | | | | |
| 704 (3 RCTs) | not serious | not serious | not serious | serious^a^ | none | ⨁⨁⨁◯ Moderate^a^ | 9/236 (3.8%) | 442/468 (94.4%) | **RR 21.49** (9.89 to 46.73) | 9/236 (3.8%) | **781 more per 1,000** (from 339 more to 1,000 more) |
| **≥1-Grade Improvement at Day 30 for HFLs** | | | | | | | | | | | |
| 704 (3 RCTs) | not serious | not serious | not serious | serious^b^ | none | ⨁⨁⨁◯ Moderate^b^ | 11/236 (4.7%) | 436/468 (93.2%) | **RR 19.17** (9.23 to 39.80) | 11/236 (4.7%) | **847 more per 1,000** (from 384 more to 1,000 more) |
| **≥1-Grade Improvement at Day 30 for LCLs** | | | | | | | | | | | |
| 704 (3 RCTs) | not serious | serious^c^ | not serious | serious^d^ | none | ⨁⨁◯◯ Low^c,d^ | 18/236 (7.6%) | 418/468 (89.3%) | **RR 11.68** (7.49 to 18.20) | 18/236 (7.6%) | **815 more per 1,000** (from 495 more to 1,000 more) |
| **≥2-Grade Improvement at Day 30 for GFLs** | | | | | | | | | | | |
| 542 (2 RCTs) | not serious | not serious | not serious | serious^e^ | none | ⨁⨁⨁◯ Moderate^e^ | 0/184 (0.0%) | 182/358 (50.8%) | **RR 94.54** (13.35 to 669.35) | 0/184 (0.0%) | **0 fewer per 1,000** (from 0 fewer to 0 fewer) |
| **≥2-grade improvement at Day 30 for HFLs** | | | | | | | | | | | |
| 542 (2 RCTs) | not serious | not serious | not serious | serious^f^ | none | ⨁⨁⨁◯ Moderate^f^ | 0/184 (0.0%) | 222/358 (62.0%) | **RR 115.04** (16.26 to 813.76) | 0/184 (0.0%) | **0 fewer per 1,000** (from 0 fewer to 0 fewer) |
| **≥2-grade improvement at Day 30 for LCLs** | | | | | | | | | | | |
| 542 (2 RCTs) | not serious | not serious | not serious | serious^g^ | none | ⨁⨁⨁◯ Moderate^g^ | 0/184 (0.0%) | 150/358 (41.9%) | **RR 76.26** (10.76 to 540.75) | 0/184 (0.0%) | **0 fewer per 1,000** (from 0 fewer to 0 fewer) |
| **Investigator assessed GAIS** | | | | | | | | | | | |
| 542 (2 RCTs) | not serious | serious^h^ | not serious | not serious | none | ⨁⨁⨁◯ Moderate^h^ | 184 | 358 | - | 184 | MD **2.29 higher** (2.11 higher to 2.46 higher) |
| **Investigator-assessed GFLs MAS 0/1 responder at Day 30** | | | | | | | | | | | |
| 543 (2 RCTs) | not serious | not serious | not serious | serious^i^ | none | ⨁⨁⨁◯ Moderate^i^ | 0/185 (0.0%) | 314/358 (87.7%) | **RR 163.62** (23.16 to 1155.94) | 0/185 (0.0%) | **0 fewer per 1,000** (from 0 fewer to 0 fewer) |
| **Investigator-assessed HFLs MAS 0/1 responder at Day 30** | | | | | | | | | | | |
| 543 (2 RCTs) | not serious | not serious | not serious | serious^j^ | none | ⨁⨁⨁◯ Moderate^j^ | 1/185 (0.5%) | 319/358 (89.1%) | **RR 103.48** (21.03 to 509.34) | 1/185 (0.5%) | **554 more per 1,000** (from 108 more to 1,000 more) |
| **Investigator-assessed LCLs MAS 0/1 responder at Day 30** | | | | | | | | | | | |
| 543 (2 RCTs) | not serious | not serious | not serious | serious^k^ | none | ⨁⨁⨁◯ Moderate^k^ | 5/185 (2.7%) | 281/358 (78.5%) | **RR 28.53** (12.01 to 67.82) | 5/185 (2.7%) | **744 more per 1,000** (from 298 more to 1,000 more) |
| **Any Adverse Events** | | | | | | | | | | | |
| 704 (3 RCTs) | not serious | not serious | not serious | serious^l^ | none | ⨁⨁⨁◯ Moderate^l^ | 84/236 (35.6%) | 192/468 (41.0%) | **RR 1.13** (0.94 to 1.37) | 84/236 (35.6%) | **46 more per 1,000** (from 21 fewer to 132 more) |
| **Headache** | | | | | | | | | | | |
| 704 (3 RCTs) | not serious | serious^m^ | not serious | serious^n^ | none | ⨁⨁◯◯ Low^m,n^ | 11/236 (4.7%) | 47/468 (10.0%) | **RR 2.05** (0.51 to 8.25) | 11/236 (4.7%) | **49 more per 1,000** (from 23 fewer to 338 more) |
| **Serious adverse events** | | | | | | | | | | | |
| 548 (2 RCTs) | not serious | not serious | not serious | serious^o^ | none | ⨁⨁⨁◯ Moderate^o^ | 5/185 (2.7%) | 1/363 (0.3%) | **RR 0.14** (0.02 to 0.85) | 5/185 (2.7%) | **23 fewer per 1,000** (from 26 fewer to 4 fewer) |

**CI:** confidence interval; **MD:** mean difference; **RR:** risk ratio

#### Explanations

a. The Imprecision domain for the outcome (≥1-Grade Improvement at Day 30 for Glabellar Frown Lines) was judged as Serious due to the extreme width of the 95% Confidence Interval (CI), which indicates a high degree of uncertainty regarding the true magnitude of the treatment effect.

b. The Imprecision domain for the outcome (≥1-Grade Improvement at Day 30 for Horizontal Forehead Lines) was marked as Serious because the estimate of the treatment effect is highly uncertain due to an extremely wide Confidence Interval (CI).

c. The inconsistency domain was marked Serious because the statistical heterogeneity is 55%. This exceeds the 50% threshold, indicating moderate inconsistency or true variation in the treatment effect across the studies.

d. The imprecision domain was marked Serious because the 95% Confidence Interval (CI) (7.49–18.20) is significantly wide for the Risk Ratio of 11.68. This wide range prevents a precise estimate of the benefit's magnitude, as the true effect could be anywhere from 7.5-fold to 18.2-fold.

e. The pooled Risk Ratio (RR) is 94.54. The 95 Confidence Interval (CI) is extremely wide, ranging from 13.35–669.35. This vast range prevents a precise conclusion about the actual magnitude of the enormous benefit.

f. The pooled Risk Ratio (RR) is 115.04. The 95\% Confidence Interval (CI) is extremely wide, ranging from 16.26–813.76. This vast range prevents a precise conclusion about the actual magnitude of the large treatment effect.

g. The pooled Risk Ratio (RR) is 76.26. The 95 Confidence Interval (CI) is extremely wide, ranging from 10.76–540.75. This vast range prevents a precise conclusion about the actual magnitude of the treatment effect. The upper bound is over 50 times the lower bound, demonstrating extreme uncertainty in the size of the benefit.

h. The statistical measure of heterogeneity is 64%. This value is above the 50% threshold, indicating high heterogeneity or true variation in the treatment effect across the two studies.

i. The pooled Risk Ratio (RR) is 163.62. The 95% Confidence Interval (CI) is extremely wide, ranging from 23.16–1155.94. This vast range prevents a precise conclusion about the actual magnitude of the large treatment effect.

j. The pooled Risk Ratio (RR) is 103.48. The 95% Confidence Interval (CI) is extremely wide, ranging from 21.03–509.34. This vast range prevents a precise conclusion about the actual magnitude of the large treatment effect, with the upper bound being over 24 times the lower bound.

k. The pooled Risk Ratio (RR) is 28.53. The 95% Confidence Interval (CI) is wide, ranging from 12.01–67.82. While this range is statistically significant, the lower bound is less than half the point estimate, showing significant uncertainty about the precise magnitude of the large benefit.

l. The pooled Risk Ratio (RR) is 1.13. The 95% Confidence Interval (CI) is 0.94–1.37. This CI crosses the line of no effect (RR). More importantly, the CI includes both the possibility of no effect and a substantial benefit or harm. Therefore, the estimate is too imprecise to rule out either a small increase in risk or no difference in risk.

m. The statistical measure of heterogeneity is 55%. This value is > 50%, indicating moderate heterogeneity across the three RCTs. This suggests genuine variability in the occurrence of headaches that is not due to chance alone

n. The pooled Risk Ratio (RR) is 2.05. The 95% Confidence Interval (CI) is extremely wide, ranging from 0.51–8.25. This CI crosses the line of no effect (RR=1). The interval includes the possibilities of a large protective effect (RR=0.51) or a large harmful effect (RR=8.25).

o. The pooled Risk Ratio (RR) is 0.14. The 95% Confidence Interval (CI) is wide, ranging from 0.02–0.85. While the CI does not cross the line of no effect (RR=1), the difference between the lower bound (0.02) and the upper bound (0.85) represents a large range of possible effects (from a 98% risk reduction to only a 15% risk reduction). Given the extremely small number of events (1 in the experimental group, 5 in the control group), the estimate is unstable and lacks the Optimal Information Size (OIS).
