# Supplementary material for: Efficacy and Safety of IncobotulinumtoxinA for the Simultaneous Treatment of Upper Facial Lines: A GRADE Assessed Systematic Review and Meta-analysis
Source: Aesthet Surg J Open Forum. 2026 May 9;8:ojag085. doi: 10.1093/asjof/ojag085 (PMC13253580; doi:10.1093/asjof/ojag085)

**Supplementary Figure 1:** PRISMA 2020 Checklist


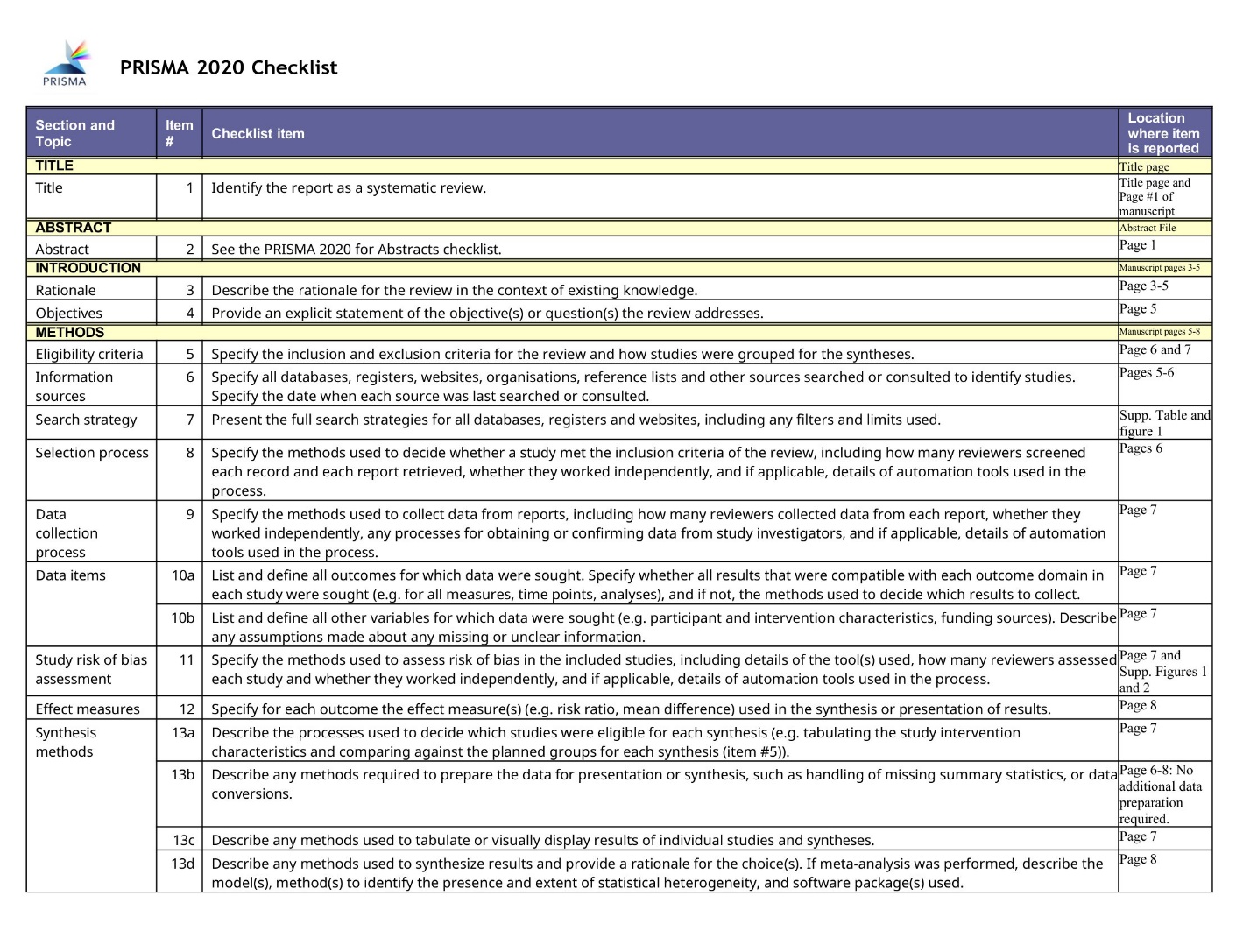

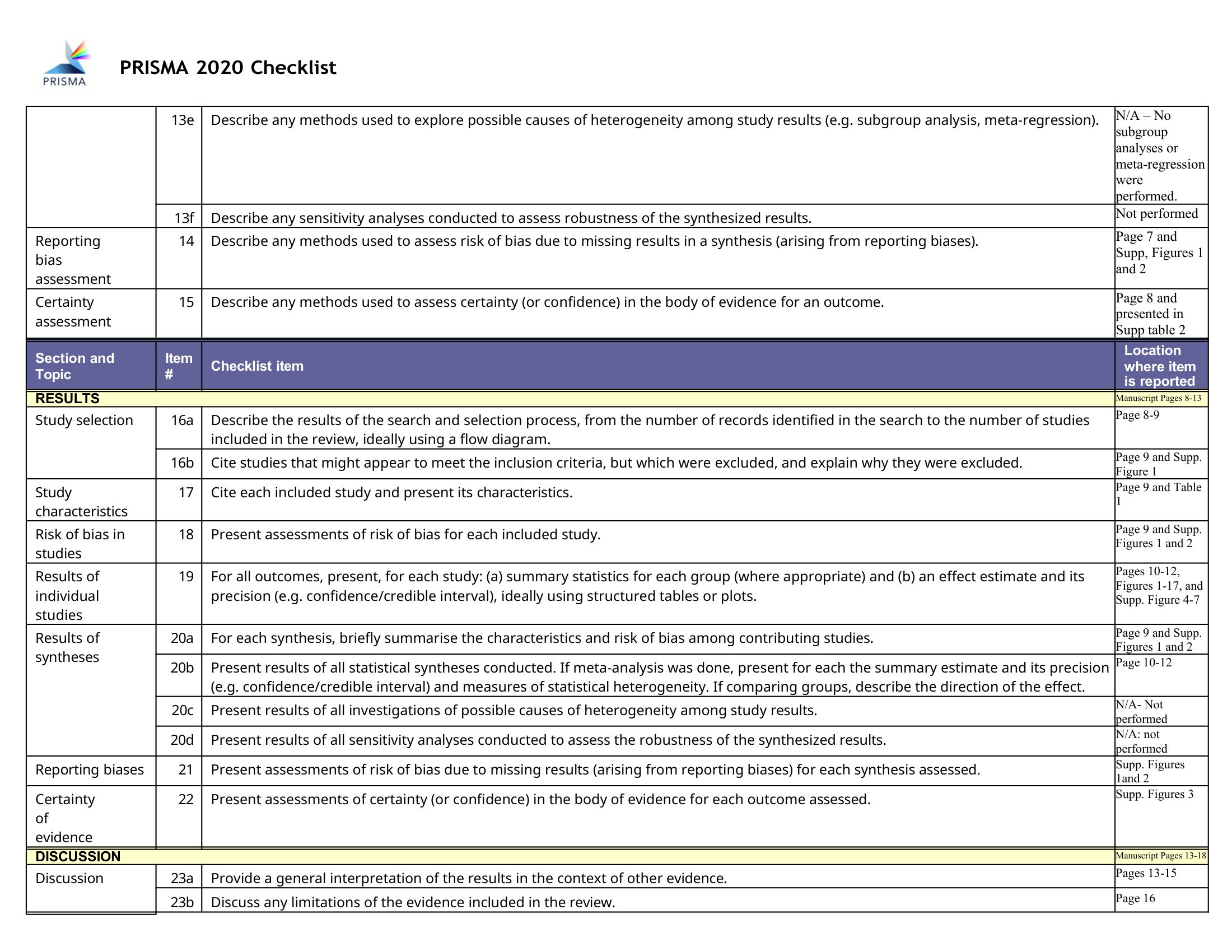


**
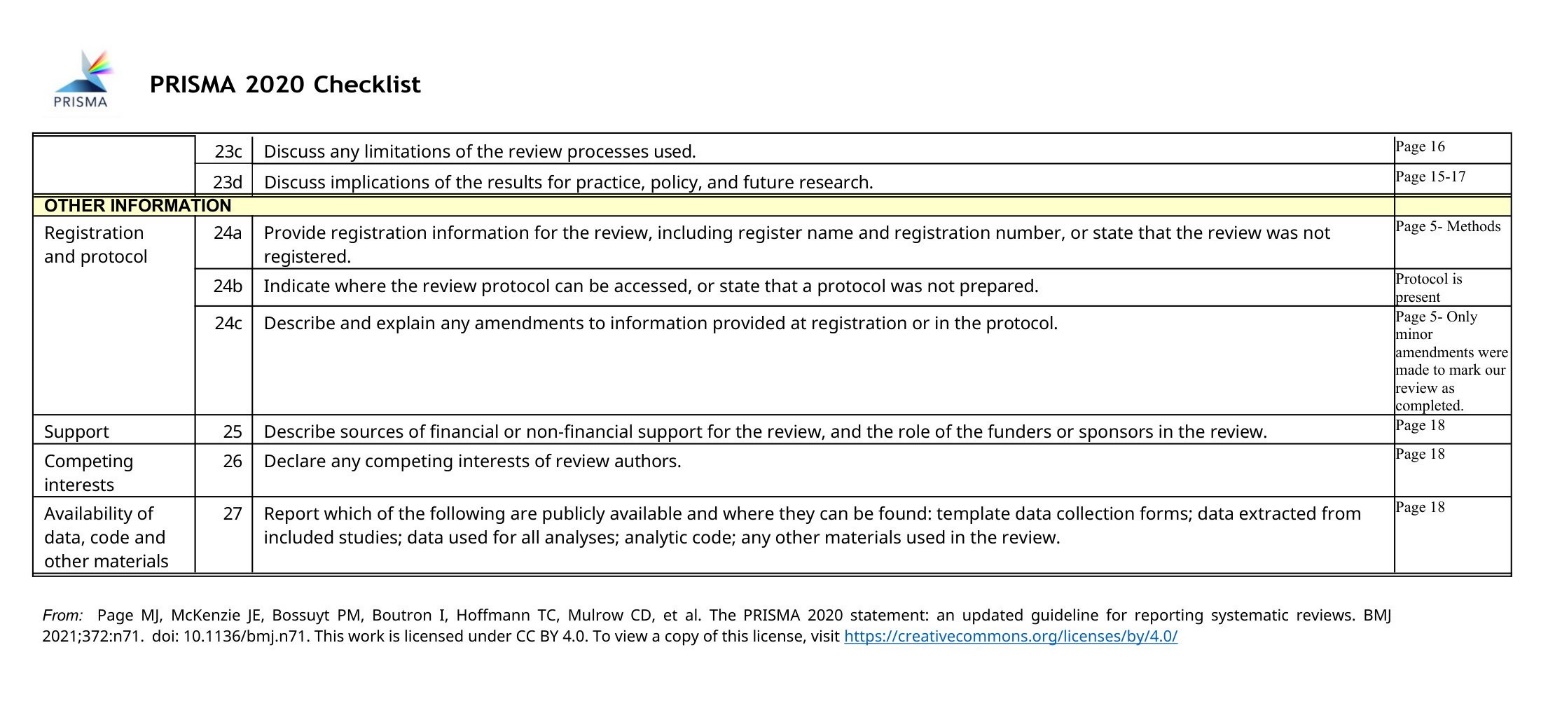
**

**Supplementary Figure 2:** Illustration of the Merz Aesthetics Scale (MAS)


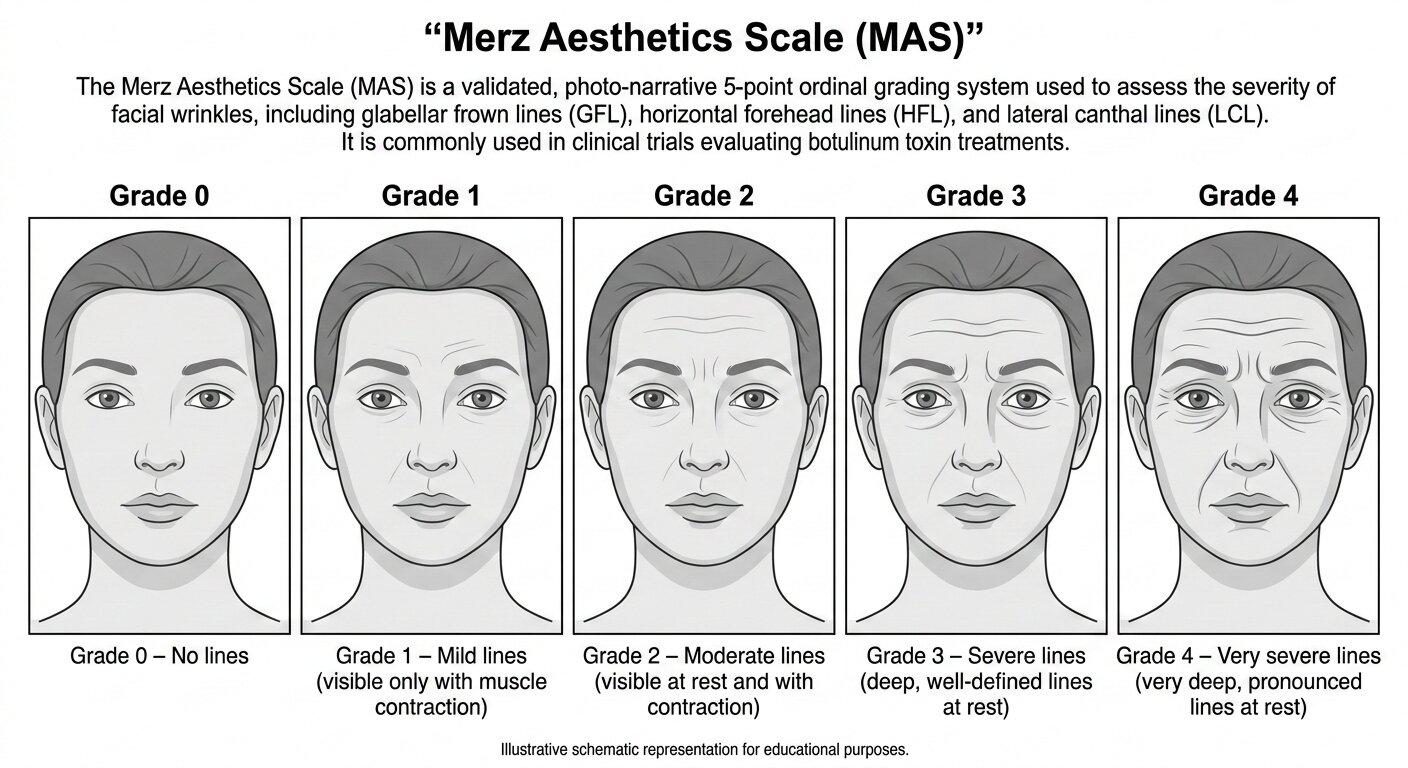


**Supplementary Figure 3:** ROB assessment results of included studies based on efficacy outcomes.


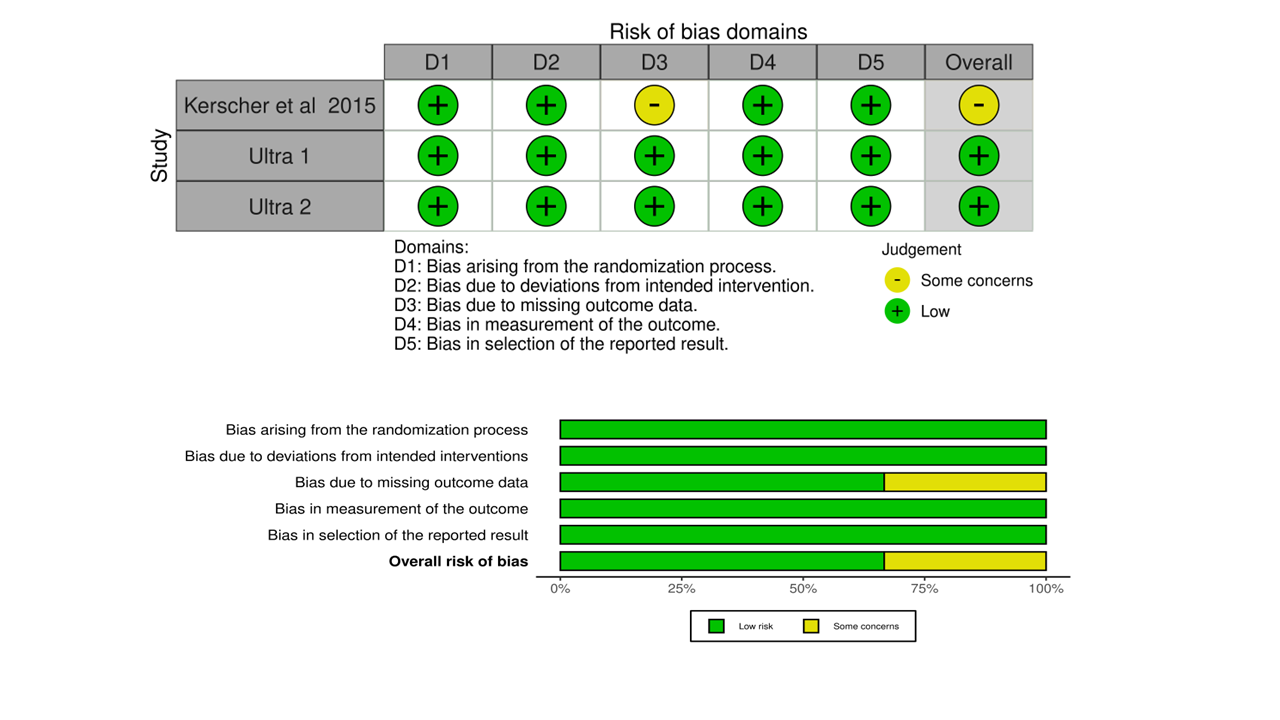


**Supplementary Figure 4:** ROB assessment results of included studies based on safety outcomes.


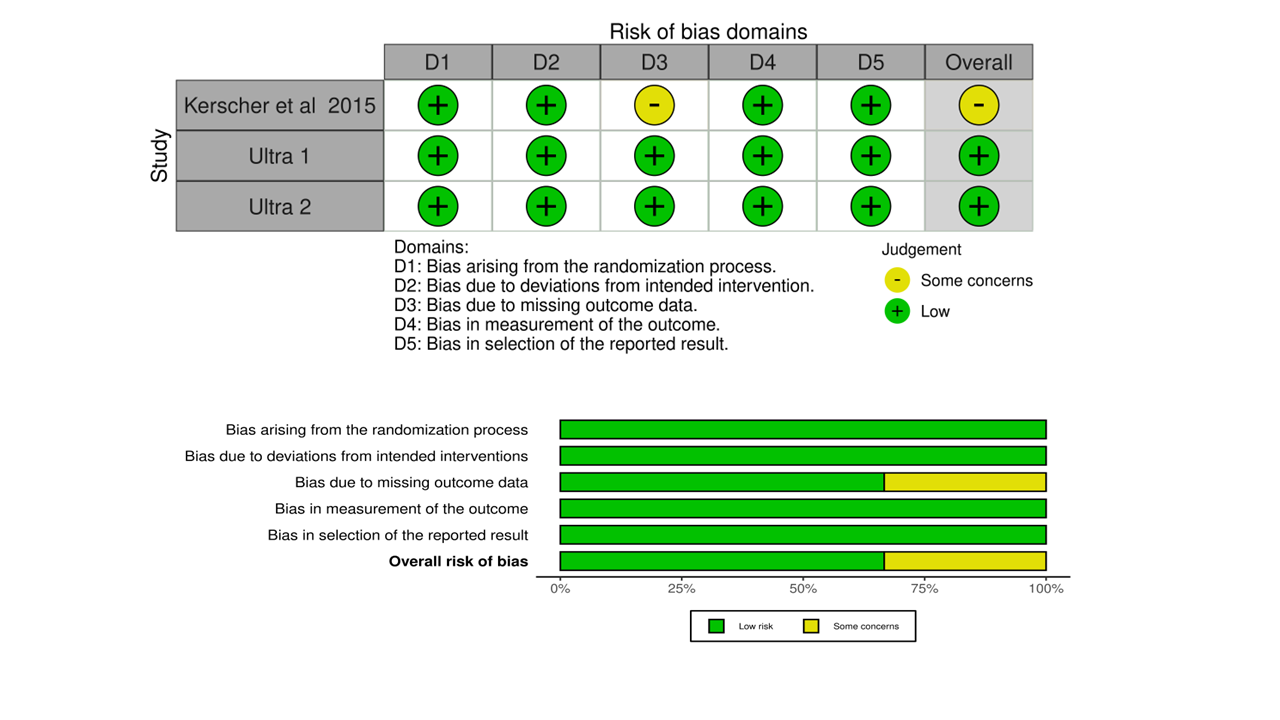


**Supplementary Figure 5:** Forest plot of the **Headache** comparing IncobotulinumtoxinA with placebo. Effect measure: risk ratio (RR) with 95% confidence intervals, analyzed using a random-effects model.


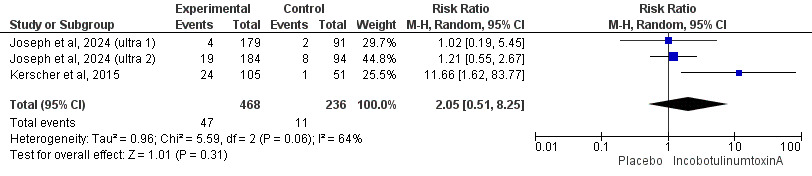


**Supplementary Figure 6:** Forest plot of the **Treatment-emergent adverse events (TEAEs)** comparing IncobotulinumtoxinA with placebo. Effect measure: risk ratio (RR) with 95% confidence intervals, analyzed using a random-effects model.


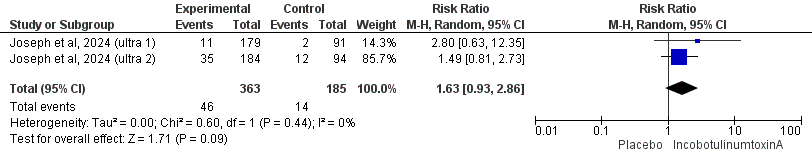


**Supplementary Figure 7:** Forest plot of the **COVID-19 related TEAEs** comparing IncobotulinumtoxinA with placebo. Effect measure: risk ratio (RR) with 95% confidence intervals, analyzed using a random-effects model.


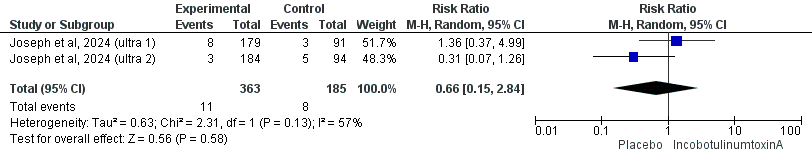


**Supplementary Figure 8:** Forest plot of the **COVID-19 infection** comparing IncobotulinumtoxinA with placebo. Effect measure: risk ratio (RR) with 95% confidence intervals, analyzed using a random-effects model.


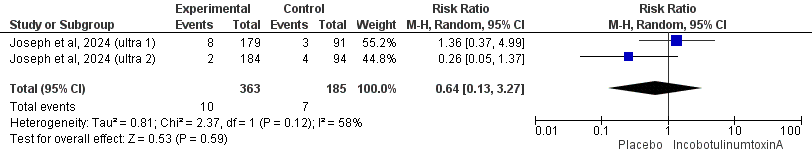


**Supplementary Figure 9:** Forest plot of the **Brow ptosis** comparing IncobotulinumtoxinA with placebo. Effect measure: risk ratio (RR) with 95% confidence intervals, analyzed using a random-effects model.


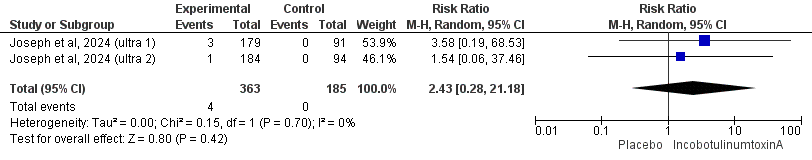

Supplement: ojag085_Supplementary_Data [file ojag085_supplementary_data.zip › Updated_Supplementary_Figures.docx]
